# Fingolimod suppresses neuronal autophagy through the mTOR/p70S6K pathway and alleviates ischemic brain damage in mice

**DOI:** 10.1371/journal.pone.0188748

**Published:** 2017-11-29

**Authors:** Xiao Li, Ming-Huan Wang, Chuan Qin, Wen-Hui Fan, Dai-Shi Tian, Jun-Li Liu

**Affiliations:** 1 Department of Neurology, Tongji Hospital, Tongji Medical College, Huazhong University of Science and Technology, Wuhan, China; 2 Department of geriatrics, Wuhan General Hospital of PLA, Wuhan, China; 3 Cancer center, Union Hospital, Tongji Medical College, Huazhong University of Science and Technology, Wuhan, China; Indian Institute of Integrative Medicine CSIR, INDIA

## Abstract

The bioactive, signaling lipid, sphingosine-1-phosphate (S1P), and its analog, fingolimod (FTY720), have previously shown neuroprotective effects against ischemic brain injury. However, the underlying mechanisms have not yet been fully clarified. The roles of autophagy in ischemic stroke are being increasingly recognized. In the present study, we sought to determine whether the S1P pathway is involved in neuronal autophagy and investigate its possible mechanisms following stroke. Interestingly, we found that FTY720 significantly attenuates infarct volumes and reduces neuronal apoptosis on days 1 and 3 post stroke, accompanied by amelioration of functional deficits. Additionally, FTY720 was found to decrease the induction of autophagosome proteins, microtubule-associated protein 1 light chain 3(LC3-II) and Beclin1, following ischemic stroke in a dose-dependent manner. Meanwhile, protein levels of the mammalian target of rapamycin (mTOR) and the 70-kDa ribosomal protein, S6 kinase1 (p70S6K), were also up-regulated in FTY720-treated animals, and the nonspecific SphK inhibitor, N,N-dimethylsphingosine (DMS), was found to cause a reverse effect. Our results indicate that modulation of the S1P signaling pathway by FTY720 could effectively decrease neuronal autophagy through the mTOR/p70S6K pathway and attenuate ischemic brain injury in mice.

## Introduction

Ischemic stroke is a devastating disease that is a major cause of death and disability, worldwide [1]. Consequently, there is a need to develop novel and effective therapies; especially, for patients with acute cerebral ischemia. Ischemic stroke triggers many detrimental cascades that mediate pathological changes including excitotoxicity, oxidative stress, inflammation, and apoptosis [2]. Recent experimental studies suggest a pivotal role of autophagy in post-ischemic changes of various organs, including the brain [3]. Although there is no debate about the existence of autophagy in cerebral ischemia, the exact function and influence of autophagy in stroke remain controversial. It has been reported that rapamycin attenuates experimental ischemic stroke damage via the activation of mitophagy [4], indicating that autophagy is beneficial for ischemia–reperfusion injury. However, increasing evidence also suggests that over-activated neuronal autophagy in cerebral-ischemic injury promotes detrimental effects [5,6].

Autophagy, which literally means “self-eating,” is a physiological degradation process that constitutively turns over cytoplasmic components in response to stress and nutrient deprivation. A proper course of autophagy in neurons is followed when normal cellular homeostasis is maintained through the elimination of damaged cellular products [7,8]. However, during stressful conditions such as nutrient deprivation or ischemia, cells require enhanced autophagy to degrade superfluous proteins and organelles to produce sufficient nutrients and energy. The initiation stage of the autophagy process requires the presence of Beclin1/Vps34/p150 protein complexes, which allows the recruitment of other proteins to form a phagophore. In the elongation phase, cytoplasmic light chain- (LC-) 3-I is converted to LC3-II, which localizes in autophagosomal membranes; the ratio of LC3-II to LC3-I is closely correlated with the number of autophagosomes [9]. Mammalian target of rapamycin (mTOR) is an evolutionarily conserved serine/threonine kinase that serves as a key modulator of autophagy in mammalian cells. The activation of mTOR promotes protein synthesis and negatively regulates autophagy by phosphorylating its downstream effectors, ribosomal protein S6 kinase beta-1 (p70S6K) and 4E Binding Protein 1(4EBP1).

Fingolimod (FTY720), an immune modulator that was recently approved for the treatment of multiple sclerosis [10,11], is rapidly phosphorylated by sphingosine kinase (SphK) 2, after administration, and subsequently assumes a structure similar to that of sphingosine-1-phosphate (S1P) [12]. Phosphorylated FTY720 acts at four S1P receptors, S1PR1, 4, 5, and to a lesser extent at S1PR3 [13], and thus exerts its biological effects through the S1P signaling pathways. N,N-dimethylsphingosine (DMS) could inhibit SphK, which is essential for S1P synthesis. There is growing evidence that FTY720 shows powerful neuroprotection on stroke outcome in the acute and long-term phases of cerebral ischemia [13–16]. It has also been reported in recent clinical trials that FTY720 can reduce brain injury and enhance functional recovery following stroke [17]; however, the exact mechanisms of FTY720 against ischemic stroke are yet to be elucidated. In tumor cells, FTY720 has been reported to induce apoptosis through the activation of autophagic cascades [18,19]. Alinari *et al* demonstrated that FTY720 enhances anticancer efficacy of milatuzumab in mantle cell lymphoma through inhibition of the autophagic pathway [20]. In the present study, we investigated whether the neuroprotective effects of FTY720, following ischemic stroke, are accompanied by the regulation of autophagic activity, and the putative role of the mTOR pathway in this process.

## Materials and methods

### 2.1. Photothrombotic ischemia model

Adult male C57/B6 mice (22–28 g) were obtained from Hunan SJA Laboratory Animal Co. Ltd., Hunan, China. The experimental procedures were performed in accordance with protocols approved by the Institutional Animal Care and Use Committee at Tongji Medical College, Huazhong University of Science and Technology. A total of 150 animals were randomly assigned to each experimental group. The photothrombotic ischemia (PT) model was developed according to our previously described method [21]. The animals were briefly anesthetized using intraperitoneal ketamine (75 mg/kg) and xylazine (20 mg/kg) injection. Using aseptic technique, cephalic skin was longitudinally incised and the periosteum was removed after the head was immobilized in the stereotactic apparatus. Following peritoneal injection of Rose Bengal (Sigma, 50 mg/kg, dissolved in saline), a photosensitive dye, animals were irradiated for 20 minutes through the intact skull, using a cold light source (KL1500LCD, Schott. 15 W, 550 nm). For illumination, a light spot 3 mm in diameter was focused on the right sensorimotor cortex (0.5 mm anterior to the bregma, and 2 mm lateral from the midline) to induce a focal stroke. Sham-operated animals underwent the same procedure, except that they received the injection of saline instead of Bengal Rose. After injury, animals were returned to their individual cages and kept warm with a heating pad. Intensive care was provided to minimize animal distress. Euthanasia was performed using an overdose of pentobarbital-containing euthanasia solution followed by cervical dislocation and decapitation. All efforts were made to minimize the number of animals used and their suffering throughout the experimental procedures.

### 2.2. Pharmacological treatments

As the previous study reported [22], mice were intraperitoneally treated with either different concentrations of FTY720, including 0.5, 1, or 2 mg/kg of FTY720 (dissolved in 0.9% sodium chloride, Cayman Chemical), the nonspecific SphK inhibitor, DMS (0.5 mg/kg, Cayman Chemical), or the mTOR inhibitor, rapamycin (0.5 mg/kg, MedChem Express), according to the experimental plan. All drugs were administered 2 hours (h) after ischemia induction and subsequently administered every day until the mice were sacrificed. Sham groups received equal volumes of saline.

### 2.3. Magnetic Resonance Imaging (MRI) examination

Mice were anesthetized on days 1 and 3 after PT to evaluate the infarct volume using a 3.0T MRI scanner (GE Healthcare, SignaHDxt) with an 8-channel head coil by an investigator blinded to the animal group. The mice’s heads were placed in an animal device with an inner diameter of 30 mm, for signal excitation and detection. The following MRI parameters were set: time to echo = 135.1 ms, repetition time = 3080 ms, field of view = 4 × 4 cm, *M* = 192 × 160, number of average = 12, thickness = 1 mm, and gap = 0.2 mm. Off-line workstation (General Electric, Advantage workstation, edition 4.4) was used to analyze the infarct volume after optimal adjustment of the contrast.

### 2.4. Behavioral assessment

The modified neurological severity scores (mNSS) test were assessed by a blinded observer on post-ischemic mice for 7 days, in each group (n = 8), as reported previously [23,24]. mNSS includes a series of neurological tests (0 to 3 points for each test): (1) spontaneous activity, (2) symmetry in limb movement, (3) forepaw outstretching, (4) climbing, (5) body proprioception, and (6) response to vibrissae touch. Neurological score was graded on a scale of 0 (normal function) to 18 (most severe impairment).

### 2.5. Immunohistochemistry staining and Terminal-Deoxynucleotidyl Transferase Mediated Nick End Labeling (TUNEL) staining

Mice were transcardially perfused with saline, followed by ice-cold 4% paraformaldehyde (PFA), under anesthesia. Brains were removed from the cranium and post-fixed for 24 h in 4% PFA and then immersed in 30% sucrose solution, until they sank. Frozen sections of brain tissues were sectioned on a Leica sliding microtome (CM1900; Leica, Wetzlar, Germany) at a thickness of 10 μm for immunohistochemistry staining and Nissl staining, and 8 μm for TUNEL staining. Slices were stored at –80°C until further use.

A series of coronal sections were selected to perform double-immunostaining. The cerebral sections were post fixed in 4% paraformaldehyde for 15 min. Fixed tissues were blocked with 10% bovine serum albumin / phosphate buffered saline (BSA/PFA) at room temperature (RT) for 1 h and incubated simultaneously with primary antibodies [anti-LC3, 1:100, Cell Signaling Technology, Beverly, MA, USA; anti-Beclin1, 1:100, Novus Biologicals, Littleton, CO, USA; anti- glial fibrillary acidic protein (GFAP), 1:200, Cell Signaling Technology, Beverly, MA, USA; anti-NeuN, 1:200, Millipore Corporation, Bedford, MA, USA] at 4°C overnight. Negative control sections were incubated with PBS instead of primary antibodies. After washing extensively with PBS, the sections were incubated at RT for 1 h with corresponding secondary antibodies and then counter-labeled with 4',6-diamidino-2-phenylindole (DAPI) for 10 min. Fluorescent signals in the penumbral area were observed with an Olympus BX-51 fluorescence microscope.

For Nissl staining, sections of identical lesions in different groups were stained with 0.1% toluidine blue, dehydrated, mounted with neutral balsam, and covered with a coverslip. Five view-fields were randomly chosen from the penumbral regions at 200× magnification under a light microscope (Olympus, Japan). Neurons with pyknotic nuclei and apoptotic morphology were excluded.

TUNEL staining was applied to determine neuronal apoptosis induced by ischemic stroke according to the manufacture’s protocol in the In Situ Apoptosis Fluorescein Detection kit (Roche Diagnostics, 12156792910). The average number of TUNEL positive neurons was counted using the ImageJ software (National Institute of Health) by an observer blinded to the treatment.

### 2.6. Western blot analysis

The procedure for western blot was performed as described previously [21]. After sacrificing the mice, the brain cortex tissue containing the injury site (2 mm long, centered at the injury site) was quickly removed and extracted in radioimmunoprecipitation (RIPA) Lysis Buffer with a protease inhibitor cocktail. Tissue lysates were then centrifuged at 12,000 ×g (4°C) for 15 min and the supernatant was collected to determine protein concentration using a bicinchoninic acid (BCA) assay kit. Fifty micrograms of protein extract from each sample was separated by sodium dodecyl sulfate polyacrylamide gel electrophoresis (SDS-PAGE) and then transferred onto a nitrocellulose membrane (0.45 μm, Millipore) using a wet transfer system. After blocking by a buffer containing 5% fat-free dry milk in tris-buffered saline (TBS), the following primary antibodies were used for western blot analysis: anti-GAPDH, 1:5000, Boster, China; rabbit anti-LC3, 1:800, Cell Signaling Technology; rabbit anti-Beclin1, 1:1000, Novus Biologicals; anti-p62, 1:1000, Abcam; rabbit anti-cleaved caspase 3, 1:1000, Cell Signaling Technology; rabbit anti-p-mTOR(ser2448), 1:500, Cell Signaling Technology; rabbit anti-mTOR, 1:1000, Cell Signaling Technology; rabbit anti-p-p70S6K(ser389), 1:500; Cell Signaling Technology; rabbit anti-p70S6K, 1:1000, Cell Signaling Technology; rabbit anti-GAPDH, 1:2000, Neomarkers. After incubation with secondary antibodies [horseradish peroxidase conjugated immunoglobulin (IgG), 1:5000, Santa Cruz, USA], the enhanced chemiluminescence (ECL) kit (Thermo Fisher Scientific, Pierce, CAN) was used to detect blots. Digital images were sequentially analyzed by ImageJ to obtain the optical densities (OD) of signals, which were semi-quantified and expressed as a ratio of the OD of the tested proteins to the OD of the control, GAPDH.

### 2.7. Transmission electron microscopy (TEM) analysis

The peri-infarct cortex surrounding the ischemia core was quickly removed and fixed with 2.5% glutaraldehyde solution, overnight at 4°C. One night later, tissues were postfixed again in 1% osmium tetroxide and dehydrated in grade dethyl alcohol. After embedding in an Epon/Araldite mixture, the samples were sectioned with an ultramicrotome (Leica, UC6) into 80-nm thick sections, and then stained in saturated uranyl acetate and lead citrate. Ultrastructural images were captured with a transmission electron microscope (Tecnai G2, FEI) at an accelerating voltage of 80 kV.

### 2.8. Statistical analysis

SPSS 19.0 software was used for statistical analysis. Student’s t test or one-way analysis of variance (ANOVA) followed by Tukey’s post-hoc test was performed to test differences between groups. All data was expressed as mean ± standard error of the mean (SEM). Statistical significance was accepted at a value of p < 0.05.

## Results

### 3.1. FTY720 alleviates neuronal apoptosis and improves functional recovery after cerebral ischemia

**Fig 1:**
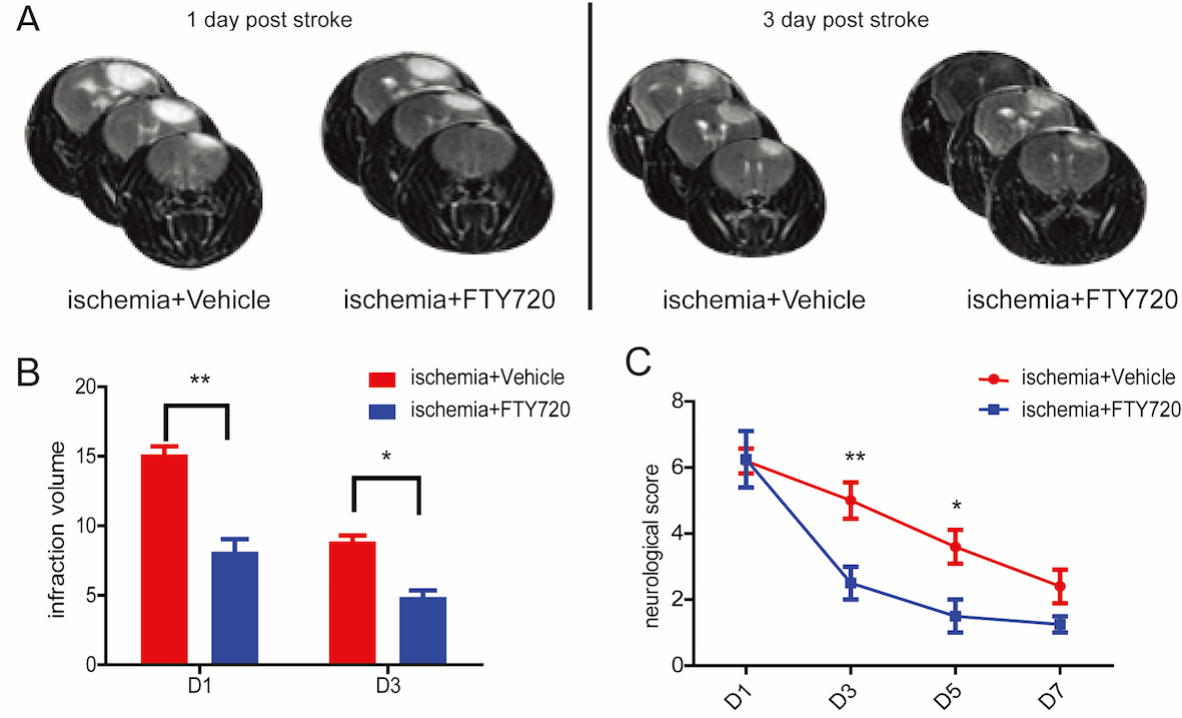
FTY720 alleviates ischemic brain damage and improves functional recovery following cerebral ischemia. **A,** Representative MRI images for the ischemia+vehicle treatment group and ischemia+FTY720 treatment group at 1 day (left) and 3 days (right) after photothrombotic ischemia. **B,** Reduced infarction volume was assessed by MRI examination after treatment with FTY720 at 1 day and 3 days post photothrombotic injury. **C,** Neurological deficiency was evaluated at 1, 3, 5, and 7 days post ischemia. Values are expressed as mean ± SEM and data were evaluated by the Student’s t test (n = 6, *p <0.05 vs. ischemia+vehicle group, **p <0.01 vs. ischemia+vehicle group).

We first tested neuroprotective effects of FTY720 following photothrombotic ischemic injury in mice. Infarct volumes by MRI detection were significantly reduced at day 1 and 3 after PT in the ischemia+FTY720 group, compared to that in the ischemia+vehicle group (Fig 1A and 1B). Furthermore, neurological scores were evaluated dynamically at day 1, 3, 5, and 7 after PT. Our results showed that the recovery of neurological deficits was accelerated by FTY720 administration (Fig 1C), indicating that the application of FTY720 could attenuate ischemia-induced brain injury.

**Fig 2:**
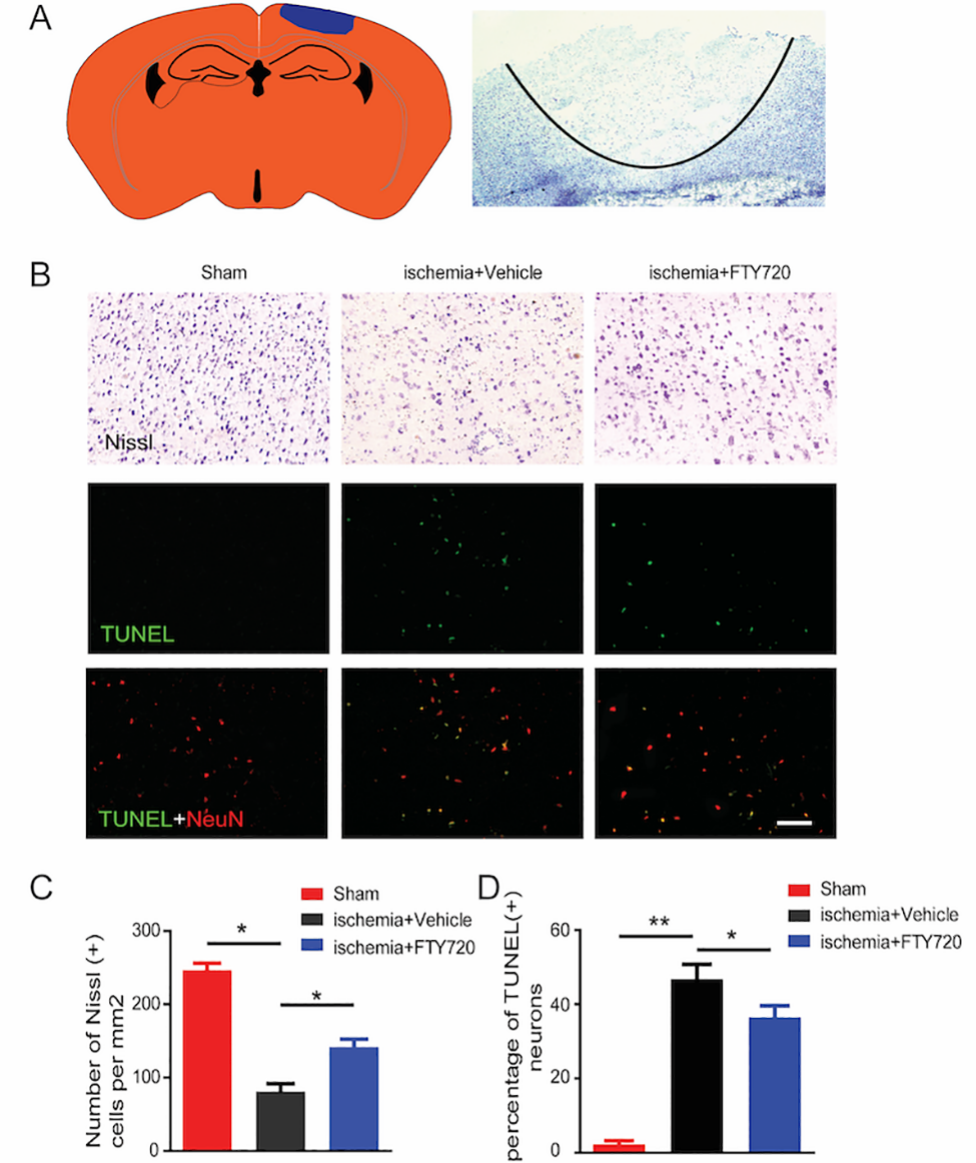
FTY720 attenuates neuronal apoptosis following ischemic stroke. **A,** Schematic diagram of the photothrombotic stroke model (left) and ischemic lesions evaluated by Nissl staining (right). **B,** Nissl staining (B1-3) and TUNEL assays (B4-9) were performed to assess effects of FTY720 on neuronal survival and apoptosis in the boundary of the ischemic lesion core at 1 day post injury. Sham mice (without ischemia induction) were also tested in parallel. The scale bar represents 50 μm. **C,** Quantification of the results from panels B1-3. **D,** Quantification of the results from panels B4-6. (n = 4 for each group, #p < 0.05 vs. sham group, *p < 0.05 vs. ischemia+vehicle group, **p < 0.01 vs. ischemia+vehicle group, one-way ANOVA followed with Tukey post hoc test).

To assess the effects of FTY720 on neuronal survival and apoptosis in the boundary zone of the ischemic lesion, Nissl staining and TUNEL assays were performed at day 1 post ischemia (Fig 2A). The number of neuronal cells with integrative and well-maintained morphology was lower in the ischemia+vehicle group than in the sham (no PT) group, based on the Nissl staining findings (Fig 2B1-2). However, cellular loss in the penumbral area was partly rescued by FTY720 administration, and was thus lower in the ischemia+FTY720 group compared to the ischemia+vehicle group (Fig 2B3 and 2C). Furthermore, double immunostaining of NeuN and TUNEL showed that TUNEL-positive neurons were obviously decreased in the ischemia+FTY720 group compared to the ischemia+vehicle group (Fig 2B4-9 and 2D). These results indicate that the neuroprotective effect of FTY720 might be associated with a reduction in neuronal apoptosis after ischemic stroke.

### 3.2. FTY720 attenuates neuronal autophagy following cerebral ischemia in a dose-dependent manner

**Fig 3:**
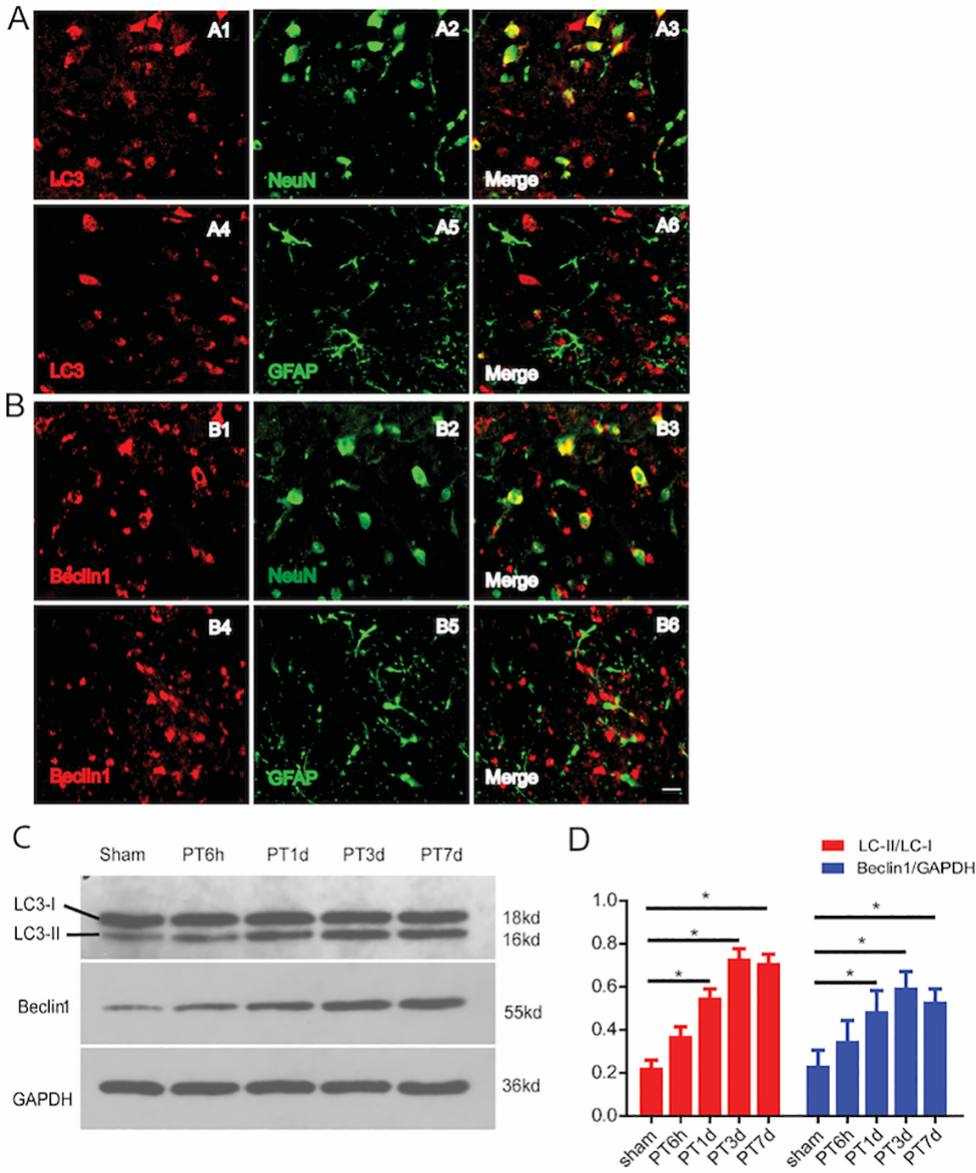
Cellular expression of LC3 and Beclin1 following ischemic stroke. **A,** Double-staining for LC3 (red) and NeuN (green) showed that numerous LC3-positive cells were colocalized within neurons in the cortex (A1-A3). However, staining against LC3 and GFAP showed that very few LC3-positive cells were colocalized with astrocytes in the lesion core surrounded areas, 1 day post stroke (A4-A6). **B,** Immunofluorescent staining against Beclin1 (red) and GFAP (green) showed that Beclin1 positive cells were mainly colocalized with neurons in the cortex instead of astrocytes (B1-B3). Scale bar represents 20 μm. **C,** The expression of LC3 and Beclin1 was assessed by western blotting at 6 h, 1 day, 3 days, and 7 days post injury. **D,** The expression of autophagosomes peaked at 3 days and then decreased slightly at 7 days. (n = 4 for each group, *p < 0.05 vs. sham group, ** p < 0.01 vs. sham group, one-way ANOVA with Tukey post hoc test).

Given that FTY720 has been reported to be involved in the autophagic process associated with anticancer efficacy of milatuzumab in mantle cell lymphoma [18,19,20], here, we investigated the potential effects of FTY720 on autophagy following ischemic stroke. To determine whether autophagy is found in ischemic brains, the expression levels of LC3 and Beclin1, two commonly used biomarkers of autophagy activation, were examined by immunofluorescence staining and western blotting at day 1 post stroke. Double-immunostaining for LC3 and NeuN indicated that numerous LC3-positive cells are mainly colocalized with neurons in the ischemic brain region; whereas, double-staining with LC3 and GFAP showed minimal colocalization with astrocytes (Fig 3A). Similar results were obtained by double-immunostaining with Beclin1, which also mainly colocalized with neurons in ischemic regions (Fig 3B). Western blotting for LC3 and Beclin1 in ischemic brain tissues at 6 h, 1 day, 3 days, and 7 days post stroke demonstrated that the ratio of LC3-II/LC-I and the expression of Beclin1 increase at 6 h post ischemia and reach their peaks at 3 days post stroke (Fig 3C and 3D). These results suggest that the process of autophagy mainly occurs in neurons instead of astrocytes in the acute phase following PT.

**Fig 4:**
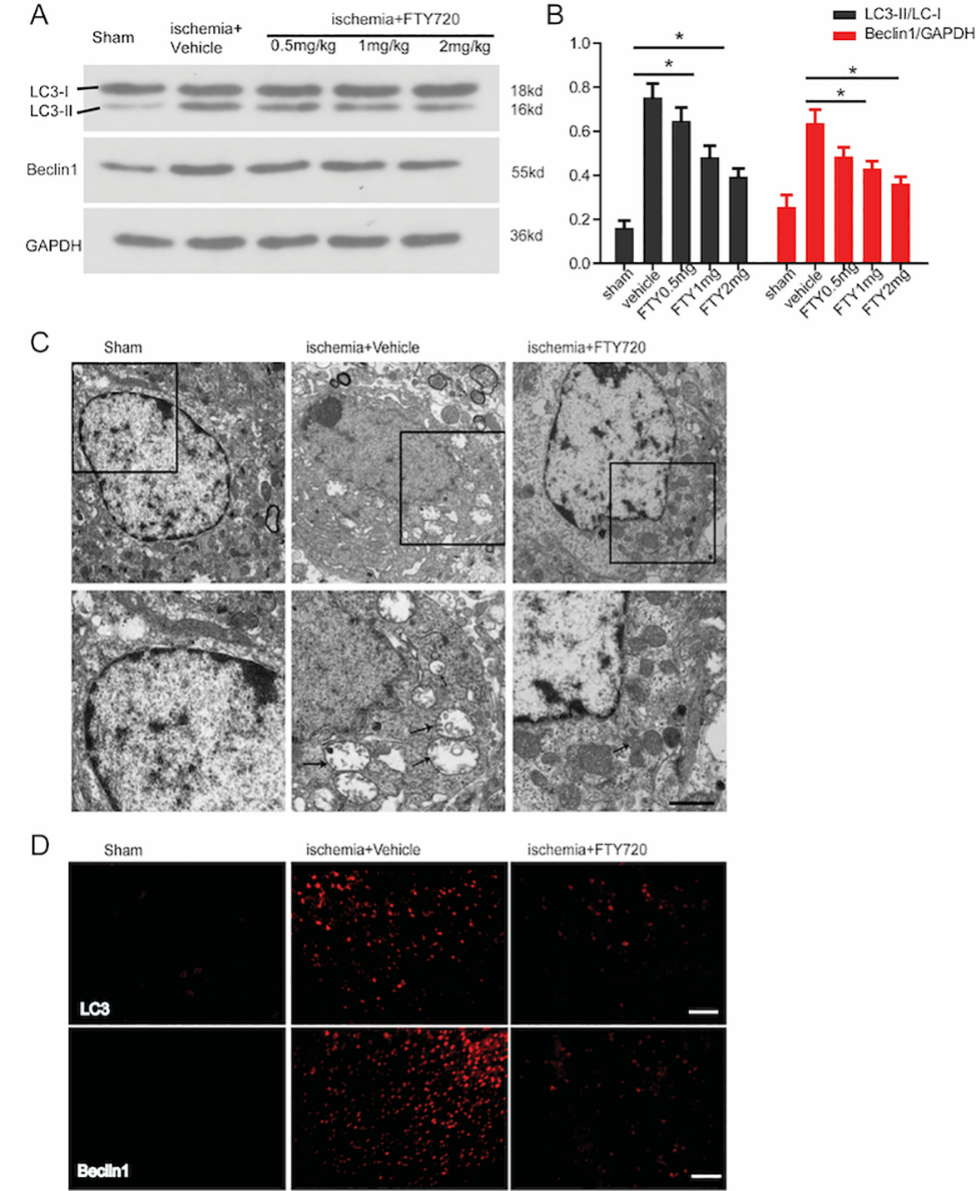
FTY720 attenuates activation of autophagy following ischemic stroke in a dose-dependent manner. **A**, The impact of increasing concentrations of FTY720 on the expression of Beclin1 and LC3 was evaluated by western blotting. Results are representative of 4 independent experiments. **B,** Quantification of the results in panel A. (n = 4 for each group, *p < 0.05 vs. sham group, **p < 0.01 vs. sham group, one-way ANOVA with Tukey post hoc test). **C,** Top, Representative electron microphotographs in neurons in the sham, ischemia+vehicle, and ischemia+FTY720 treated groups at 3 days post photothrombic ischemic stroke (A1-A3). Higher magnification images of the square area in A1-A3 showed abundant lysosomes and autophagosomes (arrows) in the ischemia+vehicle group, which was reversed by treatment with FTY720 (A4-A6). D. Representative images of LC3 and Beclin1 staining in sham treated, ischemia+vehicle, and ischemia+FTY720 groups.

**Fig 5:**
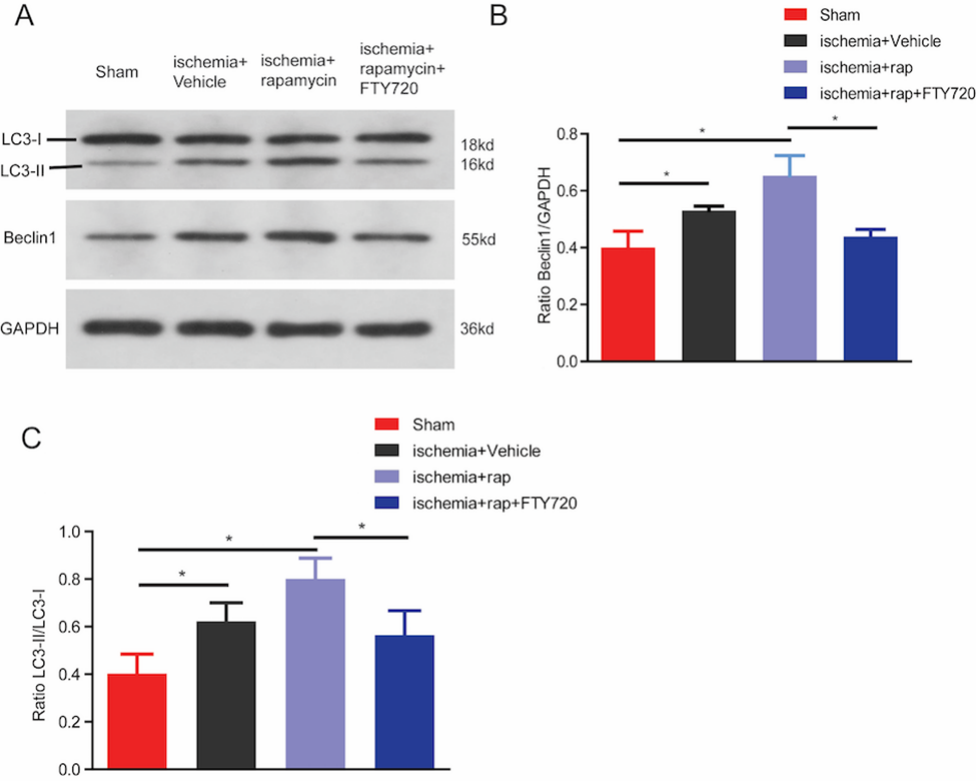
FTY720 reverses activation of autophagy induced by mTOR inhibitor, rapamycin. A, The impact of FTY720 and rapamycin on the expression of Beclin1 and LC3 was evaluated by western blotting. Results are representative of 4 independent experiments. **B,** Quantification of the results in panel A. (n = 4 for each group, *p < 0.05 vs. sham group, *p < 0.01 vs. ischemia+rapamycin group, one-way ANOVA with Tukey post hoc test).

To determine whether FTY720 could affect autophagy post ischemia, Western blot analysis was performed for brain tissues from the ischemic boundary zone in the presence of different concentrations of FTY720 for 3 days. The increased LC3-II/LC3-I ratio and Beclin1 expression after ischemia were partly reversed by FTY720 treatment in a dose-dependent manner (Fig 4A and 4B). To further confirm our findings, we performed the TEM technique. Neurons in the sham group appeared to have intact karyotheca, endoplasmic reticulum, and mitochondria. However, the morphology of neurons in the ischemia+vehicle group was disrupted, with lysed mitochondria, lysosomes and autophagosomes. FTY720 treatment reduced the formation of autophagosomes and lysosomes and alleviated neuronal injury (Fig 4C). In line with the result of TEM, the immunostaining results of autophagosomal markers indicated that the number of LC3 and Beclin1 positive cells decreased in the ischemia+FTY720 group (Fig 4D). Moreover, increased LC3-II/LC3-I ratio and Beclin1 expression after PT was further enhanced by the administration of rapamycin, an agonist of autophagy. This synergic effect of the autophagic process could be abolished by FTY720 treatment (Fig 5A–5C). These results confirmed that FTY720 could suppress autophagy activation following ischemic stroke.

**Fig 6:**
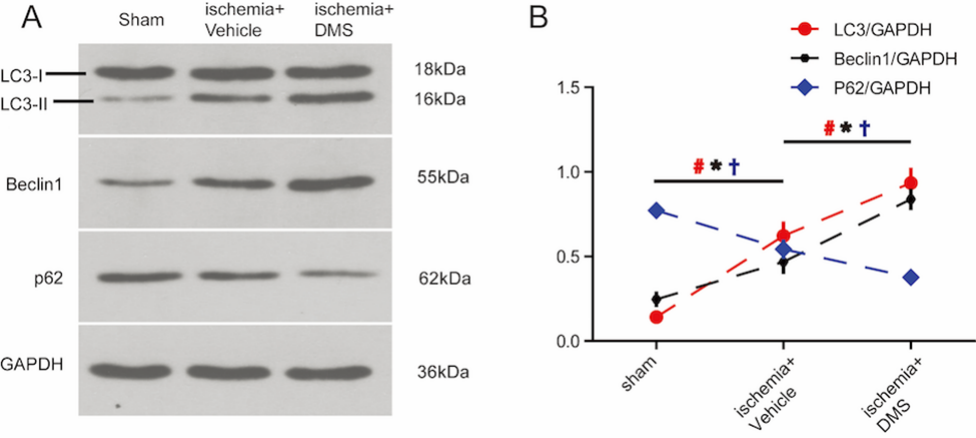
The S1P signaling pathway regulates activation of autophagy, post stroke. **A,** Impact of SphK inhibitor, DMS, on expression of Beclin1, LC3, and the main autophagy substrate, p62, detected by western blotting. Results are representative of 4 independent experiments. **B,** Quantification of the results from panel A (n = 4 for each group, # p < 0.05 vs. sham group, ** p < 0.01 vs. ischemia+vehicle group, one-way ANOVA with Tukey post hoc test).

To verify the role of the S1P/SphK pathway in autophagy following ischemic injury, we evaluated the possible effects of the SphK inhibitor, DMS, on the autophagic process following stroke. Consistent with the aforementioned results, the expression levels of Beclin1 and LC3 significantly increased in the ischemia+vehicle group, compared to expression levels in the sham group. Furthermore, the expression of the main autophagic substrate, p62, also reduced after ischemia (Fig 6A). DMS also caused a further increase in the expression of LC3-II and Beclin1 and a decrease in the expression of p62, which further confirmed the role of S1P/SphK signaling in ischemic brain injury (Fig 6A and 6B).

### 3.3. FTY720 suppresses autophagy activity after ischemic stroke by activating mTOR/p70S6 signaling pathway

**Fig 7:**
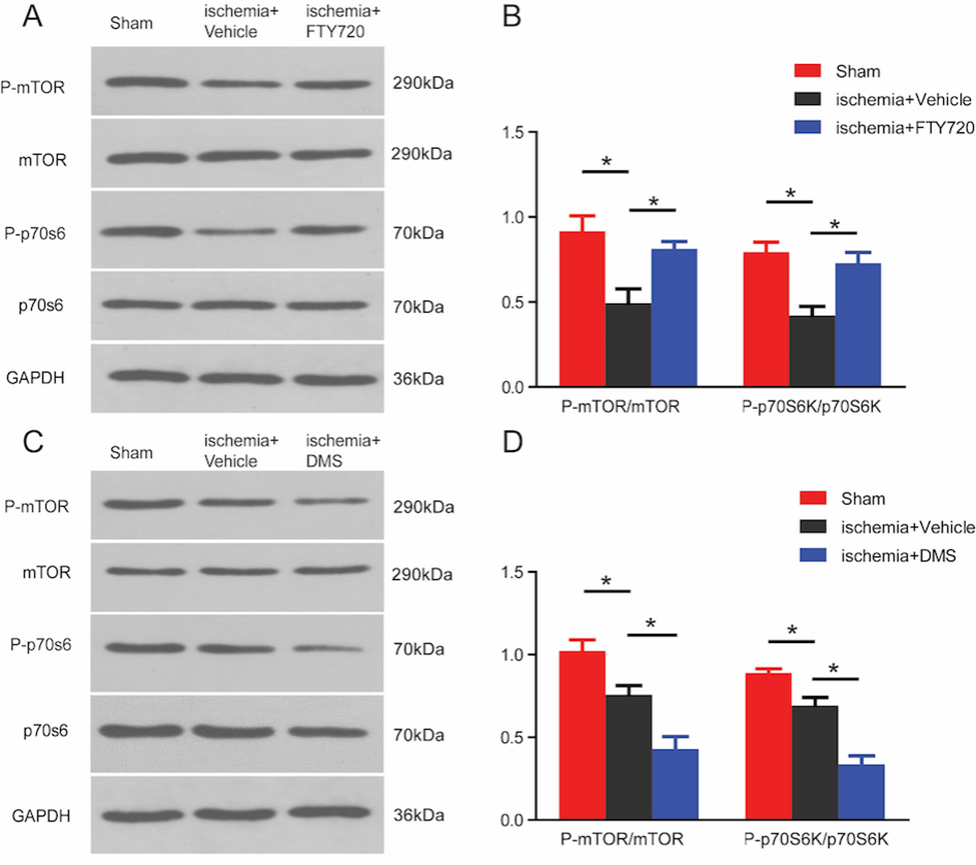
The S1P signaling pathway modulates the mTOR/p70S6K pathway following photothrombic ischemic stroke (PT). **A,** Representative western blots of p-mTOR/t-mTOR and its downstream target p-p70S6K/t-p70S6K at 3days after PT. **B,** Quantification of the results from panel A. **C,** The impact of the SphK inhibitor, DMS, on the expression of the mTOR/p70S6K signalling pathway was assessed by western blotting. **D,** Quantification of the results in panel C. (n = 4 for each group, # p < 0.05 vs. sham group,*p < 0.05 vs. ischemia+vehicle group, one-way ANOVA with Tukey post hoc test).

Since mTOR/p70S6K signaling pathway is known to play a pivotal role in regulating autophagy activity, we evaluated whether FTY720 might inhibit autophagy by activating mTOR pathway after ischemic stroke. Our results demonstrated that the expression of phosphorylated mTOR (p-mTOR) was significantly reduced at 3 days post injury, while this reduction was reversed by treatment with FTY720 (Fig 7A and 7B). A corresponding decline in the expression of p70S6K, the downstream substrate for p-mTOR, was also observed after ischemia and was reversed by FTY720 administration, which was consistent with the possibility that FTY720 suppresses autophagy by activating the mTOR/p70S6K signaling pathway following stroke.

To verify the role of the S1P/SphK pathway in modulating the activation of the mTOR/ p70S6K pathway during ischemia, we treated mice with the SphK inhibitor, DMS, for 3 days. Our results demonstrated that DMS decreased the enhanced expression of p-mTOR and p70S6K activated by cerebral ischemia (Fig 7C and 7D), which confirmed that S1P/SphK signaling could modulate the mTOR/p70S6K pathway during autophagy.

## Discussion

Our results in this study showed that S1P analog, FTY720, attenuates cerebral infarction, decreases neuronal apoptosis, and improves neurological deficits in acute PT, which is consistent with results of several previous studies [15,16,22,25]. In addition, we found that FTY720 significantly reduces the number of autophagosomes and attenuates autophagic activity in a dose-dependent manner. Furthermore, FTY720 could reverse the enhanced effects of mTOR inhibitor, rapamycin, on autophagy following stroke. However, the SphK inhibitor, DMS, increases the number of autophagosomes with a corresponding reduction in the expression of the main autophagy substrate, p62, which implies that the S1P/SphK pathway is involved in the process of autophagic activation following ischemic stroke. In the present study, we concluded that FTY720 could effectively decrease neuronal autophagy and attenuate ischemic brain injury through the mTOR/p70S6K pathway. To the best of our knowledge, it is a novel finding that has never been reported previously.

Although it is inappropriate to conclude arbitrarily that the neuroprotective effects of FTY720 are due to modulation of neuronal autophagy and not due to FTY720 mediated-immunosuppression, we speculate that over-activated neuronal autophagy plays a vital role in acute ischemic stroke and is associated with the loss of neuronal cells. The biological roles of S1P and its catabolic enzyme (SK, SPL) in autophagy are increasingly being recognized [26]. It has been reported that in a neuron model of Huntington’s disease, SK1 enhances autophagic flux and S1P-metabolizing enzymes decrease this flux. Furthermore, the expression of a dominant-negative form of SK1 inhibits autophagosome synthesis; this result identifies the S1P pathway as a novel regulator of neuronal autophagy in neurodegenerative disorders [27]. Mitroia *et al* also described that a reduction of autolysosomes and a change in LC3 distribution are found when SGPL1 (an S1P metabolizing enzyme) is genetically and pharmacologically inhibited in cultured neurons [28]. These results indicate that the S1P signaling pathway is vigorously involved in modulating the process of neuronal autophagy. Further studies would be helpful to elucidate the exact relationship between the modulation of S1P and its metabolizing enzymes, and neuronal autophagy.

It has been reported that FTY720 exerts neuroprotective effect by the induction of lymphocytopenia and a concomitant reduction of intravascular thrombo-inflammation [22,29]. A reduction of reactive astrogliosis has also been observed in FTY720-treated neurons [30]. Here, we showed that FTY720 could rescue ischemic, dying neurons directly and alleviate ischemic brain injury with the suppression of autophagic activity. It is suggested that modulation of autophagy may contribute to the protective functions of FTY720. In this study, neurons exhibited strong LC3 and Beclin 1 positive staining in the peri-ischemic cortex, indicating that the activation of autophagy could occur in neurons of ischemic brains. There was a slight discrepancy between our findings and those of a few previous studies, which described the activation of autophagy in injured astrocytes, induced by oxygen-glucose deprivation and permanent middle cerebral artery occlusion [31]. We observed the activation of autophagy in ischemic, injured neurons following PT; however, reactive astrogliosis was more evident at 7 days in the PT model. Thus, the discrepancy might be due to the different time points of observation and differences in stroke models.

It is known that the activation of autophagy can be regulated by the mTOR signal pathway, which is in turn essential for the regulation of cell viability, differentiation, transcription, actin cytoskeletal organization, and autophagy through its downstream target, p70S6K [32]. Several reports have demonstrated that S1P could induce mTOR activation in several cancer cell lines associated with fibroblasts and vascular smooth muscle cells [33–35]. The S1P1-mTOR axis is crucial for the differentiation of Th1 cells and iTregs [35,36]. It means that S1P may exert it biological function via the mTOR pathway. To further test the mechanism of FTY720 in attenuating autophagy, we evaluated the activation of the mTOR/p70S6K signaling pathway. As expected, the pathologic process of ischemic brain injury was accompanied by a decrease in p-mTOR (Ser2448) and p-p70S6K. However, this phenomenon was reversed by FTY720. Conversely, the mTOR/p70S6K signaling pathway was significantly attenuated by the SphK inhibitor, DMS, suggesting that FTY720 inhibits the over-activation of the autophagic pathway via mTOR signaling. These results corroborate the protective action of the lipid-signaling mediator, FTY720, in our experimental stroke model. It should be noted that though mTOR is an important regulator of autophagy, m-TOR-independent regulation of autophagy in neurons exists as well. Thus, FTY720 may modulate neuronal autophagy through pathways other than the mTOR/p70S6K pathway.

It has been reported that the S1P pathway may be a novel regulator of neuronal autophagy in neurodegenerative diseases [30]. As a pleiotropic lysophospholipid mediator, S1P enhances cell growth and survival through cellular responses such as suppression of apoptosis [37], calcium mobilization [38], increased cell migration [39], and mitogenesis [40]. Hence, S1P may activate signaling pathways such as the extracellular-signal-regulated kinase 1/2, calcium levels, hypoxia inducible factor-1, and protein kinase B (Akt/PKB) pathways [41–43], which are known to modulate autophagy through the mTOR signaling pathway. Besides, S1P may protect cerebral injury by preventing cell apoptosis through Bcl-2 activation [44]. This is consistent with our findings that FTY720 alleviates neuronal apoptosis following ischemic stroke. As an apoptosis-inhibiting factor, Bcl-2 inhibits the autophagy pathway by associatin with Beclin1. After associating with Bcl-2, Beclin1 escapes out of the p150/vps34 complex and thereby reduces the occurrence of autophagy. Under the condition of nutrient deficiency in the cell, Bcl-2 can inhibit Beclin1 to decrease the level of autophagy, and reduce ischemic cell death and injury of brain tissue caused by hypoxia. This may be a possible mechanism for the modulation of S1P in autophagy.

Neuroprotection via autophagy blockade in cerebral ischemia has also been reported in other studies. In a model of repeated cerebral ischemia-reperfusion injury, Xu et al [45] reported that L-3-n-Butylphthalide could reduce the LC3-II/LC3-I ratio and decrease the expression of beclin-1 messenger ribonucleic acid (mRNA) through modulation of the Akt/mTOR signaling pathway, and finally alleviate learning and memory deficits. It should be noted that although FTY720 alleviates neuronal autophagy and exerts neuroprotection following ischemic stroke in the present study, we cannot assume that autophagy is absolutely detrimental for recovery following ischemia. Whether the role of autophagy is beneficial or deleterious following ischemic stress largely depends on the condition of the intracellular stress of autophagic activation and the elaborate machinery of cellular autophagy [3]. On one hand, autophagy causes energy depletion, DNA fragmentation, apoptotic signaling pathway activation, and severe damage in the intracellular components of the ischemic stroke model. On the other hand, autophagy could also be a part of integrated prosurvival signaling, which includes the PI3K-Akt-mTOR axis, and might be crucial for ischemic preconditioning [46]. Moreover, various types of experimental models and assays for monitoring autophagy result in different conclusions [46]. Importantly, there are no absolute criteria for evaluating autophagy activation that applies to every situation. Therefore, further experiments will be necessary to specify the role of autophagy in different models of ischemic brain damage.

## Conclusion

FTY720 alleviates ischemic brain damage through the blockade of neuronal autophagic processes via modulation of the mTOR/p70S6K pathway.
